# Charmonium and *e*^+^*e*^−^ pair photoproduction at mid-rapidity in ultra-peripheral Pb–Pb collisions at $\sqrt{s_{\mathrm{NN}}} = 2.76\ \mbox{TeV}$

**DOI:** 10.1140/epjc/s10052-013-2617-1

**Published:** 2013-11-09

**Authors:** The ALICE Collaboration, E. Abbas, B. Abelev, J. Adam, D. Adamová, A. M. Adare, M. M. Aggarwal, G. Aglieri Rinella, M. Agnello, A. G. Agocs, A. Agostinelli, Z. Ahammed, N. Ahmad, A. Ahmad Masoodi, I. Ahmed, S. A. Ahn, S. U. Ahn, I. Aimo, M. Ajaz, A. Akindinov, D. Aleksandrov, B. Alessandro, D. Alexandre, A. Alici, A. Alkin, E. Almaráz Aviña, J. Alme, T. Alt, V. Altini, S. Altinpinar, I. Altsybeev, C. Andrei, A. Andronic, V. Anguelov, J. Anielski, C. Anson, T. Antičić, F. Antinori, P. Antonioli, L. Aphecetche, H. Appelshäuser, N. Arbor, S. Arcelli, A. Arend, N. Armesto, R. Arnaldi, T. Aronsson, I. C. Arsene, M. Arslandok, A. Asryan, A. Augustinus, R. Averbeck, T. C. Awes, J. Äystö, M. D. Azmi, M. Bach, A. Badalà, Y. W. Baek, R. Bailhache, R. Bala, A. Baldisseri, F. Baltasar Dos Santos Pedrosa, J. Bán, R. C. Baral, R. Barbera, F. Barile, G. G. Barnaföldi, L. S. Barnby, V. Barret, J. Bartke, M. Basile, N. Bastid, S. Basu, B. Bathen, G. Batigne, B. Batyunya, P. C. Batzing, C. Baumann, I. G. Bearden, H. Beck, N. K. Behera, I. Belikov, F. Bellini, R. Bellwied, E. Belmont-Moreno, G. Bencedi, S. Beole, I. Berceanu, A. Bercuci, Y. Berdnikov, D. Berenyi, A. A. E. Bergognon, R. A. Bertens, D. Berzano, L. Betev, A. Bhasin, A. K. Bhati, J. Bhom, L. Bianchi, N. Bianchi, C. Bianchin, J. Bielčík, J. Bielčíková, A. Bilandzic, S. Bjelogrlic, F. Blanco, F. Blanco, D. Blau, C. Blume, M. Boccioli, S. Böttger, A. Bogdanov, H. Bøggild, M. Bogolyubsky, L. Boldizsár, M. Bombara, J. Book, H. Borel, A. Borissov, F. Bossú, M. Botje, E. Botta, E. Braidot, P. Braun-Munzinger, M. Bregant, T. Breitner, T. A. Broker, T. A. Browning, M. Broz, R. Brun, E. Bruna, G. E. Bruno, D. Budnikov, H. Buesching, S. Bufalino, P. Buncic, O. Busch, Z. Buthelezi, D. Caffarri, X. Cai, H. Caines, E. Calvo Villar, P. Camerini, V. Canoa Roman, G. Cara Romeo, W. Carena, F. Carena, N. Carlin Filho, F. Carminati, A. Casanova Díaz, J. Castillo Castellanos, J. F. Castillo Hernandez, E. A. R. Casula, V. Catanescu, C. Cavicchioli, C. Ceballos Sanchez, J. Cepila, P. Cerello, B. Chang, S. Chapeland, J. L. Charvet, S. Chattopadhyay, S. Chattopadhyay, M. Cherney, C. Cheshkov, B. Cheynis, V. Chibante Barroso, D. D. Chinellato, P. Chochula, M. Chojnacki, S. Choudhury, P. Christakoglou, C. H. Christensen, P. Christiansen, T. Chujo, S. U. Chung, C. Cicalo, L. Cifarelli, F. Cindolo, J. Cleymans, F. Colamaria, D. Colella, A. Collu, G. Conesa Balbastre, Z. Conesa del Valle, M. E. Connors, G. Contin, J. G. Contreras, T. M. Cormier, Y. Corrales Morales, P. Cortese, I. Cortés Maldonado, M. R. Cosentino, F. Costa, M. E. Cotallo, E. Crescio, P. Crochet, E. Cruz Alaniz, R. Cruz Albino, E. Cuautle, L. Cunqueiro, A. Dainese, R. Dang, A. Danu, K. Das, I. Das, S. Das, D. Das, S. Dash, A. Dash, S. De, G. O. V. de Barros, A. De Caro, G. de Cataldo, J. de Cuveland, A. De Falco, D. De Gruttola, H. Delagrange, A. Deloff, N. De Marco, E. Dénes, S. De Pasquale, A. Deppman, G. D Erasmo, R. de Rooij, M. A. Diaz Corchero, D. Di Bari, T. Dietel, C. Di Giglio, S. Di Liberto, A. Di Mauro, P. Di Nezza, R. Divià, Ø. Djuvsland, A. Dobrin, T. Dobrowolski, B. Dönigus, O. Dordic, A. K. Dubey, A. Dubla, L. Ducroux, P. Dupieux, A. K. Dutta Majumdar, D. Elia, D. Emschermann, H. Engel, B. Erazmus, H. A. Erdal, D. Eschweiler, B. Espagnon, M. Estienne, S. Esumi, D. Evans, S. Evdokimov, G. Eyyubova, D. Fabris, J. Faivre, D. Falchieri, A. Fantoni, M. Fasel, D. Fehlker, L. Feldkamp, D. Felea, A. Feliciello, B. Fenton-Olsen, G. Feofilov, A. Fernández Téllez, A. Ferretti, A. Festanti, J. Figiel, M. A. S. Figueredo, S. Filchagin, D. Finogeev, F. M. Fionda, E. M. Fiore, E. Floratos, M. Floris, S. Foertsch, P. Foka, S. Fokin, E. Fragiacomo, A. Francescon, U. Frankenfeld, U. Fuchs, C. Furget, M. Fusco Girard, J. J. Gaardhøje, M. Gagliardi, A. Gago, M. Gallio, D. R. Gangadharan, P. Ganoti, C. Garabatos, E. Garcia-Solis, C. Gargiulo, I. Garishvili, J. Gerhard, M. Germain, C. Geuna, M. Gheata, A. Gheata, B. Ghidini, P. Ghosh, P. Gianotti, P. Giubellino, E. Gladysz-Dziadus, P. Glässel, R. Gomez, E. G. Ferreiro, L. H. González-Trueba, P. González-Zamora, S. Gorbunov, A. Goswami, S. Gotovac, L. K. Graczykowski, R. Grajcarek, A. Grelli, C. Grigoras, A. Grigoras, V. Grigoriev, A. Grigoryan, S. Grigoryan, B. Grinyov, N. Grion, P. Gros, J. F. Grosse-Oetringhaus, J.-Y. Grossiord, R. Grosso, F. Guber, R. Guernane, B. Guerzoni, M. Guilbaud, K. Gulbrandsen, H. Gulkanyan, T. Gunji, A. Gupta, R. Gupta, R. Haake, Ø. Haaland, C. Hadjidakis, M. Haiduc, H. Hamagaki, G. Hamar, B. H. Han, L. D. Hanratty, A. Hansen, Z. Harmanová-Tóthová, J. W. Harris, M. Hartig, A. Harton, D. Hatzifotiadou, S. Hayashi, A. Hayrapetyan, S. T. Heckel, M. Heide, H. Helstrup, A. Herghelegiu, G. Herrera Corral, N. Herrmann, B. A. Hess, K. F. Hetland, B. Hicks, B. Hippolyte, Y. Hori, P. Hristov, I. Hřivnáčová, M. Huang, T. J. Humanic, D. S. Hwang, R. Ichou, R. Ilkaev, I. Ilkiv, M. Inaba, E. Incani, G. M. Innocenti, P. G. Innocenti, M. Ippolitov, M. Irfan, C. Ivan, M. Ivanov, A. Ivanov, V. Ivanov, O. Ivanytskyi, A. Jachołkowski, P. M. Jacobs, C. Jahnke, H. J. Jang, M. A. Janik, P. H. S. Y. Jayarathna, S. Jena, D. M. Jha, R. T. Jimenez Bustamante, P. G. Jones, H. Jung, A. Jusko, A. B. Kaidalov, S. Kalcher, P. Kaliňák, T. Kalliokoski, A. Kalweit, J. H. Kang, V. Kaplin, S. Kar, A. Karasu Uysal, O. Karavichev, T. Karavicheva, E. Karpechev, A. Kazantsev, U. Kebschull, R. Keidel, B. Ketzer, M. M. Khan, P. Khan, S. A. Khan, K. H. Khan, A. Khanzadeev, Y. Kharlov, B. Kileng, M. Kim, T. Kim, B. Kim, S. Kim, M. Kim, D. J. Kim, J. S. Kim, J. H. Kim, D. W. Kim, S. Kirsch, I. Kisel, S. Kiselev, A. Kisiel, J. L. Klay, J. Klein, C. Klein-Bösing, M. Kliemant, A. Kluge, M. L. Knichel, A. G. Knospe, M. K. Köhler, T. Kollegger, A. Kolojvari, M. Kompaniets, V. Kondratiev, N. Kondratyeva, A. Konevskikh, V. Kovalenko, M. Kowalski, S. Kox, G. Koyithatta Meethaleveedu, J. Kral, I. Králik, F. Kramer, A. Kravčáková, M. Krelina, M. Kretz, M. Krivda, F. Krizek, M. Krus, E. Kryshen, M. Krzewicki, V. Kucera, Y. Kucheriaev, T. Kugathasan, C. Kuhn, P. G. Kuijer, I. Kulakov, J. Kumar, P. Kurashvili, A. Kurepin, A. B. Kurepin, A. Kuryakin, V. Kushpil, S. Kushpil, H. Kvaerno, M. J. Kweon, Y. Kwon, P. Ladrón de Guevara, C. Lagana Fernandes, I. Lakomov, R. Langoy, S. L. La Pointe, C. Lara, A. Lardeux, P. La Rocca, R. Lea, M. Lechman, S. C. Lee, G. R. Lee, I. Legrand, J. Lehnert, R. C. Lemmon, M. Lenhardt, V. Lenti, H. León, M. Leoncino, I. León Monzón, P. Lévai, S. Li, J. Lien, R. Lietava, S. Lindal, V. Lindenstruth, C. Lippmann, M. A. Lisa, H. M. Ljunggren, D. F. Lodato, P. I. Loenne, V. R. Loggins, V. Loginov, D. Lohner, C. Loizides, K. K. Loo, X. Lopez, E. López Torres, G. Løvhøiden, X.-G. Lu, P. Luettig, M. Lunardon, J. Luo, G. Luparello, C. Luzzi, R. Ma, K. Ma, D. M. Madagodahettige-Don, A. Maevskaya, M. Mager, D. P. Mahapatra, A. Maire, M. Malaev, I. Maldonado Cervantes, L. Malinina, D. Mal’Kevich, P. Malzacher, A. Mamonov, L. Manceau, L. Mangotra, V. Manko, F. Manso, V. Manzari, Y. Mao, M. Marchisone, J. Mareš, G. V. Margagliotti, A. Margotti, A. Marín, C. Markert, M. Marquard, I. Martashvili, N. A. Martin, P. Martinengo, M. I. Martínez, G. Martínez García, Y. Martynov, A. Mas, S. Masciocchi, M. Masera, A. Masoni, L. Massacrier, A. Mastroserio, A. Matyja, C. Mayer, J. Mazer, R. Mazumder, M. A. Mazzoni, F. Meddi, A. Menchaca-Rocha, J. Mercado Pérez, M. Meres, Y. Miake, K. Mikhaylov, L. Milano, J. Milosevic, A. Mischke, A. N. Mishra, D. Miśkowiec, C. Mitu, S. Mizuno, J. Mlynarz, B. Mohanty, L. Molnar, L. Montaño Zetina, M. Monteno, E. Montes, T. Moon, M. Morando, D. A. Moreira De Godoy, S. Moretto, A. Morreale, A. Morsch, V. Muccifora, E. Mudnic, S. Muhuri, M. Mukherjee, H. Müller, M. G. Munhoz, S. Murray, L. Musa, J. Musinsky, B. K. Nandi, R. Nania, E. Nappi, C. Nattrass, T. K. Nayak, S. Nazarenko, A. Nedosekin, M. Nicassio, M. Niculescu, B. S. Nielsen, T. Niida, S. Nikolaev, V. Nikolic, S. Nikulin, V. Nikulin, B. S. Nilsen, M. S. Nilsson, F. Noferini, P. Nomokonov, G. Nooren, A. Nyanin, A. Nyatha, C. Nygaard, J. Nystrand, A. Ochirov, H. Oeschler, S. Oh, S. K. Oh, J. Oleniacz, A. C. Oliveira Da Silva, J. Onderwaater, C. Oppedisano, A. Ortiz Velasquez, A. Oskarsson, P. Ostrowski, J. Otwinowski, K. Oyama, K. Ozawa, Y. Pachmayer, M. Pachr, F. Padilla, P. Pagano, G. Paić, F. Painke, C. Pajares, S. K. Pal, A. Palaha, A. Palmeri, V. Papikyan, G. S. Pappalardo, W. J. Park, A. Passfeld, D. I. Patalakha, V. Paticchio, B. Paul, A. Pavlinov, T. Pawlak, T. Peitzmann, H. Pereira Da Costa, E. Pereira De Oliveira Filho, D. Peresunko, C. E. Pérez Lara, D. Perrino, W. Peryt, A. Pesci, Y. Pestov, V. Petráček, M. Petran, M. Petris, P. Petrov, M. Petrovici, C. Petta, S. Piano, M. Pikna, P. Pillot, O. Pinazza, L. Pinsky, N. Pitz, D. B. Piyarathna, M. Planinic, M. Płoskoń, J. Pluta, T. Pocheptsov, S. Pochybova, P. L. M. Podesta-Lerma, M. G. Poghosyan, K. Polák, B. Polichtchouk, N. Poljak, A. Pop, S. Porteboeuf-Houssais, V. Pospíšil, B. Potukuchi, S. K. Prasad, R. Preghenella, F. Prino, C. A. Pruneau, I. Pshenichnov, G. Puddu, V. Punin, J. Putschke, H. Qvigstad, A. Rachevski, A. Rademakers, T. S. Räihä, J. Rak, A. Rakotozafindrabe, L. Ramello, S. Raniwala, R. Raniwala, S. S. Räsänen, B. T. Rascanu, D. Rathee, W. Rauch, A. W. Rauf, V. Razazi, K. F. Read, J. S. Real, K. Redlich, R. J. Reed, A. Rehman, P. Reichelt, M. Reicher, R. Renfordt, A. R. Reolon, A. Reshetin, F. Rettig, J.-P. Revol, K. Reygers, L. Riccati, R. A. Ricci, T. Richert, M. Richter, P. Riedler, W. Riegler, F. Riggi, A. Rivetti, M. Rodríguez Cahuantzi, A. Rodriguez Manso, K. Røed, E. Rogochaya, D. Rohr, D. Röhrich, R. Romita, F. Ronchetti, P. Rosnet, S. Rossegger, A. Rossi, P. Roy, C. Roy, A. J. Rubio Montero, R. Rui, R. Russo, E. Ryabinkin, A. Rybicki, S. Sadovsky, K. Šafařík, R. Sahoo, P. K. Sahu, J. Saini, H. Sakaguchi, S. Sakai, D. Sakata, C. A. Salgado, J. Salzwedel, S. Sambyal, V. Samsonov, X. Sanchez Castro, L. Šándor, A. Sandoval, M. Sano, G. Santagati, R. Santoro, J. Sarkamo, D. Sarkar, E. Scapparone, F. Scarlassara, R. P. Scharenberg, C. Schiaua, R. Schicker, H. R. Schmidt, C. Schmidt, S. Schuchmann, J. Schukraft, T. Schuster, Y. Schutz, K. Schwarz, K. Schweda, G. Scioli, E. Scomparin, R. Scott, P. A. Scott, G. Segato, I. Selyuzhenkov, S. Senyukov, J. Seo, S. Serci, E. Serradilla, A. Sevcenco, A. Shabetai, G. Shabratova, R. Shahoyan, S. Sharma, N. Sharma, S. Rohni, K. Shigaki, K. Shtejer, Y. Sibiriak, E. Sicking, S. Siddhanta, T. Siemiarczuk, D. Silvermyr, C. Silvestre, G. Simatovic, G. Simonetti, R. Singaraju, R. Singh, S. Singha, V. Singhal, T. Sinha, B. C. Sinha, B. Sitar, M. Sitta, T. B. Skaali, K. Skjerdal, R. Smakal, N. Smirnov, R. J. M. Snellings, C. Søgaard, R. Soltz, M. Song, J. Song, C. Soos, F. Soramel, I. Sputowska, M. Spyropoulou-Stassinaki, B. K. Srivastava, J. Stachel, I. Stan, G. Stefanek, M. Steinpreis, E. Stenlund, G. Steyn, J. H. Stiller, D. Stocco, M. Stolpovskiy, P. Strmen, A. A. P. Suaide, M. A. Subieta Vásquez, T. Sugitate, C. Suire, M. Suleymanov, R. Sultanov, M. Šumbera, T. Susa, T. J. M. Symons, A. Szanto de Toledo, I. Szarka, A. Szczepankiewicz, M. Szymański, J. Takahashi, M. A. Tangaro, J. D. Tapia Takaki, A. Tarantola Peloni, A. Tarazona Martinez, A. Tauro, G. Tejeda Muñoz, A. Telesca, A. Ter Minasyan, C. Terrevoli, J. Thäder, D. Thomas, R. Tieulent, A. R. Timmins, D. Tlusty, A. Toia, H. Torii, L. Toscano, V. Trubnikov, D. Truesdale, W. H. Trzaska, T. Tsuji, A. Tumkin, R. Turrisi, T. S. Tveter, J. Ulery, K. Ullaland, J. Ulrich, A. Uras, G. M. Urciuoli, G. L. Usai, M. Vajzer, M. Vala, L. Valencia Palomo, S. Vallero, P. Vande Vyvre, J. W. Van Hoorne, M. van Leeuwen, L. Vannucci, A. Vargas, R. Varma, M. Vasileiou, A. Vasiliev, V. Vechernin, M. Veldhoen, M. Venaruzzo, E. Vercellin, S. Vergara, R. Vernet, M. Verweij, L. Vickovic, G. Viesti, J. Viinikainen, Z. Vilakazi, O. Villalobos Baillie, Y. Vinogradov, L. Vinogradov, A. Vinogradov, T. Virgili, Y. P. Viyogi, A. Vodopyanov, M. A. Völkl, S. Voloshin, K. Voloshin, G. Volpe, B. von Haller, I. Vorobyev, D. Vranic, J. Vrláková, B. Vulpescu, A. Vyushin, V. Wagner, B. Wagner, R. Wan, Y. Wang, Y. Wang, M. Wang, K. Watanabe, M. Weber, J. P. Wessels, U. Westerhoff, J. Wiechula, J. Wikne, M. Wilde, G. Wilk, M. C. S. Williams, B. Windelband, C. G. Yaldo, Y. Yamaguchi, S. Yang, P. Yang, H. Yang, S. Yasnopolskiy, J. Yi, Z. Yin, I.-K. Yoo, J. Yoon, X. Yuan, I. Yushmanov, V. Zaccolo, C. Zach, C. Zampolli, S. Zaporozhets, A. Zarochentsev, P. Závada, N. Zaviyalov, H. Zbroszczyk, P. Zelnicek, I. S. Zgura, M. Zhalov, Y. Zhang, H. Zhang, X. Zhang, D. Zhou, Y. Zhou, F. Zhou, H. Zhu, J. Zhu, X. Zhu, J. Zhu, A. Zichichi, A. Zimmermann, G. Zinovjev, Y. Zoccarato, M. Zynovyev, M. Zyzak

**Affiliations:** 1 CERN, 1211 Geneva 23, Switzerland; 2 Academy of Scientific Research and Technology (ASRT), Cairo, Egypt; 3 A. I. Alikhanyan National Science Laboratory (Yerevan Physics Institute) Foundation, Yerevan, Armenia; 4 Benemérita Universidad Autónoma de Puebla, Puebla, Mexico; 5 Bogolyubov Institute for Theoretical Physics, Kiev, Ukraine; 6 Department of Physics and Centre for Astroparticle Physics and Space Science (CAPSS), Bose Institute, Kolkata, India; 7 Budker Institute for Nuclear Physics, Novosibirsk, Russia; 8 California Polytechnic State University, San Luis Obispo, California United States; 9 Central China Normal University, Wuhan, China; 10 Centre de Calcul de l’IN2P3, Villeurbanne, France; 11 Centro de Aplicaciones Tecnológicas y Desarrollo Nuclear (CEADEN), Havana, Cuba; 12 Centro de Investigaciones Energéticas Medioambientales y Tecnológicas (CIEMAT), Madrid, Spain; 13 Centro de Investigación y de Estudios Avanzados (CINVESTAV), Mexico City and Mérida, Mexico; 14 Centro Fermi - Museo Storico della Fisica e Centro Studi e Ricerche “Enrico Fermi”, Rome, Italy; 15 Chicago State University, Chicago, United States; 16 Commissariat à l’Energie Atomique, IRFU, Saclay, France; 17 COMSATS Institute of Information Technology (CIIT), Islamabad, Pakistan; 18 Departamento de Física de Partículas and IGFAE, Universidad de Santiago de Compostela, Santiago de Compostela, Spain; 19 Department of Physics, Aligarh Muslim University, Aligarh, India; 20 Department of Physics and Technology, University of Bergen, Bergen, Norway; 21 Department of Physics, Ohio State University, Columbus, Ohio United States; 22 Department of Physics, Sejong University, Seoul, South Korea; 23 Department of Physics, University of Oslo, Oslo, Norway; 24 Dipartimento di Fisica dell’Università and Sezione INFN, Trieste, Italy; 25 Dipartimento di Fisica dell’Università and Sezione INFN, Cagliari, Italy; 26 Dipartimento di Fisica dell’Università and Sezione INFN, Turin, Italy; 27 Dipartimento di Fisica dell’Università ‘La Sapienza’ and Sezione INFN, Rome, Italy; 28 Dipartimento di Fisica e Astronomia dell’Università and Sezione INFN, Catania, Italy; 29 Dipartimento di Fisica e Astronomia dell’Università and Sezione INFN, Bologna, Italy; 30 Dipartimento di Fisica e Astronomia dell’Università and Sezione INFN, Padova, Italy; 31 Dipartimento di Fisica ‘E.R. Caianiello’ dell’Università and Gruppo Collegato INFN, Salerno, Italy; 32 Dipartimento di Scienze e Innovazione Tecnologica dell’Università del Piemonte Orientale and Gruppo Collegato INFN, Alessandria, Italy; 33 Dipartimento Interateneo di Fisica ‘M. Merlin’ and Sezione INFN, Bari, Italy; 34 Division of Experimental High Energy Physics, University of Lund, Lund, Sweden; 35 European Organization for Nuclear Research (CERN), Geneva, Switzerland; 36 Fachhochschule Köln, Köln, Germany; 37 Faculty of Engineering, Bergen University College, Bergen, Norway; 38 Faculty of Mathematics, Physics and Informatics, Comenius University, Bratislava, Slovakia; 39 Faculty of Nuclear Sciences and Physical Engineering, Czech Technical University in Prague, Prague, Czech Republic; 40 Faculty of Science, P.J. Šafárik University, Košice, Slovakia; 41 Frankfurt Institute for Advanced Studies, Johann Wolfgang Goethe-Universität Frankfurt, Frankfurt, Germany; 42 Gangneung-Wonju National University, Gangneung, South Korea; 43 Department of Physics, Gauhati University, Guwahati, India; 44 Helsinki Institute of Physics (HIP) and University of Jyväskylä, Jyväskylä, Finland; 45 Hiroshima University, Hiroshima, Japan; 46 Indian Institute of Technology Bombay (IIT), Mumbai, India; 47 Indian Institute of Technology Indore (IITI), Indore, India; 48 Institut de Physique Nucléaire d’Orsay (IPNO), Université Paris-Sud, CNRS-IN2P3, Orsay, France; 49 Institute for High Energy Physics, Protvino, Russia; 50 Institute for Nuclear Research, Academy of Sciences, Moscow, Russia; 51 Nikhef, National Institute for Subatomic Physics and Institute for Subatomic Physics of Utrecht University, Utrecht, Netherlands; 52 Institute for Theoretical and Experimental Physics, Moscow, Russia; 53 Institute of Experimental Physics, Slovak Academy of Sciences, Košice, Slovakia; 54 Institute of Physics, Bhubaneswar, India; 55 Institute of Physics, Academy of Sciences of the Czech Republic, Prague, Czech Republic; 56 Institute of Space Sciences (ISS), Bucharest, Romania; 57 Institut für Informatik, Johann Wolfgang Goethe-Universität Frankfurt, Frankfurt, Germany; 58 Institut für Kernphysik, Johann Wolfgang Goethe-Universität Frankfurt, Frankfurt, Germany; 59 Institut für Kernphysik, Technische Universität Darmstadt, Darmstadt, Germany; 60 Institut für Kernphysik, Westfälische Wilhelms-Universität Münster, Münster, Germany; 61 Instituto de Ciencias Nucleares, Universidad Nacional Autónoma de México, Mexico City, Mexico; 62 Instituto de Física, Universidad Nacional Autónoma de México, Mexico City, Mexico; 63 Institut Pluridisciplinaire Hubert Curien (IPHC), Université de Strasbourg, CNRS-IN2P3, Strasbourg, France; 64 Joint Institute for Nuclear Research (JINR), Dubna, Russia; 65 Kirchhoff-Institut für Physik, Ruprecht-Karls-Universität Heidelberg, Heidelberg, Germany; 66 Korea Institute of Science and Technology Information, Daejeon, South Korea; 67 KTO Karatay University, Konya, Turkey; 68 Laboratoire de Physique Corpusculaire (LPC), Clermont Université, Université Blaise Pascal, CNRS–IN2P3, Clermont-Ferrand, France; 69 Laboratoire de Physique Subatomique et de Cosmologie (LPSC), Université Joseph Fourier, CNRS-IN2P3, Institut Polytechnique de Grenoble, Grenoble, France; 70 Laboratori Nazionali di Frascati, INFN, Frascati, Italy; 71 Laboratori Nazionali di Legnaro, INFN, Legnaro, Italy; 72 Lawrence Berkeley National Laboratory, Berkeley, California United States; 73 Lawrence Livermore National Laboratory, Livermore, California United States; 74 Moscow Engineering Physics Institute, Moscow, Russia; 75 National Centre for Nuclear Studies, Warsaw, Poland; 76 National Institute for Physics and Nuclear Engineering, Bucharest, Romania; 77 National Institute of Science Education and Research, Bhubaneswar, India; 78 Niels Bohr Institute, University of Copenhagen, Copenhagen, Denmark; 79 Nikhef, National Institute for Subatomic Physics, Amsterdam, Netherlands; 80 Nuclear Physics Institute, Academy of Sciences of the Czech Republic, Řež u Prahy, Czech Republic; 81 Oak Ridge National Laboratory, Oak Ridge, Tennessee United States; 82 Petersburg Nuclear Physics Institute, Gatchina, Russia; 83 Physics Department, Creighton University, Omaha, Nebraska United States; 84 Physics Department, Panjab University, Chandigarh, India; 85 Physics Department, University of Athens, Athens, Greece; 86 Physics Department, University of Cape Town and iThemba LABS, National Research Foundation, Somerset West, South Africa; 87 Physics Department, University of Jammu, Jammu, India; 88 Physics Department, University of Rajasthan, Jaipur, India; 89 Physikalisches Institut, Ruprecht-Karls-Universität Heidelberg, Heidelberg, Germany; 90 Politecnico di Torino, Turin, Italy; 91 Purdue University, West Lafayette, Indiana United States; 92 Pusan National University, Pusan, South Korea; 93 Research Division and ExtreMe Matter Institute EMMI, GSI Helmholtzzentrum für Schwerionenforschung, Darmstadt, Germany; 94 Rudjer Bošković Institute, Zagreb, Croatia; 95 Russian Federal Nuclear Center (VNIIEF), Sarov, Russia; 96 Russian Research Centre Kurchatov Institute, Moscow, Russia; 97 Saha Institute of Nuclear Physics, Kolkata, India; 98 School of Physics and Astronomy, University of Birmingham, Birmingham, United Kingdom; 99 Sección Física, Departamento de Ciencias, Pontificia Universidad Católica del Perú, Lima, Peru; 100 Sezione INFN, Catania, Italy; 101 Sezione INFN, Turin, Italy; 102 Sezione INFN, Padova, Italy; 103 Sezione INFN, Bologna, Italy; 104 Sezione INFN, Cagliari, Italy; 105 Sezione INFN, Trieste, Italy; 106 Sezione INFN, Bari, Italy; 107 Sezione INFN, Rome, Italy; 108 Nuclear Physics Group, STFC Daresbury Laboratory, Daresbury, United Kingdom; 109 SUBATECH, Ecole des Mines de Nantes, Université de Nantes, CNRS-IN2P3, Nantes, France; 110 Suranaree University of Technology, Nakhon Ratchasima, Thailand; 111 Technical University of Split FESB, Split, Croatia; 112 Technische Universität München, Munich, Germany; 113 The Henryk Niewodniczanski Institute of Nuclear Physics, Polish Academy of Sciences, Cracow, Poland; 114 Physics Department, The University of Texas at Austin, Austin, TX United States; 115 Universidad Autónoma de Sinaloa, Culiacán, Mexico; 116 Universidade de São Paulo (USP), São Paulo, Brazil; 117 Universidade Estadual de Campinas (UNICAMP), Campinas, Brazil; 118 Université de Lyon, Université Lyon 1, CNRS/IN2P3, IPN-Lyon, Villeurbanne, France; 119 University of Houston, Houston, Texas United States; 120 University of Technology and Austrian Academy of Sciences, Vienna, Austria; 121 University of Tennessee, Knoxville, Tennessee United States; 122 University of Tokyo, Tokyo, Japan; 123 University of Tsukuba, Tsukuba, Japan; 124 Eberhard Karls Universität Tübingen, Tübingen, Germany; 125 Variable Energy Cyclotron Centre, Kolkata, India; 126 Vestfold University College, Tonsberg, Norway; 127 V. Fock Institute for Physics, St. Petersburg State University, St. Petersburg, Russia; 128 Warsaw University of Technology, Warsaw, Poland; 129 Wayne State University, Detroit, Michigan United States; 130 Wigner Research Centre for Physics, Hungarian Academy of Sciences, Budapest, Hungary; 131 Yale University, New Haven, Connecticut United States; 132 Yildiz Technical University, Istanbul, Turkey; 133 Yonsei University, Seoul, South Korea; 134 Zentrum für Technologietransfer und Telekommunikation (ZTT), Fachhochschule Worms, Worms, Germany

## Abstract

The ALICE Collaboration at the LHC has measured the J/*ψ* and *ψ*′ photoproduction at mid-rapidity in ultra-peripheral Pb–Pb collisions at $\sqrt{s_{\mathrm{NN}}}=2.76~\mathrm{TeV}$.

The charmonium is identified via its leptonic decay for events where the hadronic activity is required to be minimal. The analysis is based on an event sample corresponding to an integrated luminosity of about 23 μb^−1^. The cross section for coherent and incoherent J/*ψ* production in the rapidity interval −0.9<*y*<0.9, are $\mathrm{d}\sigma_{\mathrm{J}/\psi}^{\mathrm{coh}}/\mathrm{d}y = 2.38^{+0.34}_{-0.24} (\mathrm{sta+sys} )~\mathrm{mb}$ and $\mathrm{d}\sigma_{\mathrm{J}/\psi}^{\mathrm{inc}}/\mathrm{d}y = 0.98^{+0.19}_{-0.17} (\mathrm{sta}+\mathrm{sys})~\mathrm{mb}$, respectively. The results are compared to theoretical models for J/*ψ* production and the coherent cross section is found to be in good agreement with those models incorporating moderate nuclear gluon shadowing at Bjorken-*x* around 10^−3^, such as EPS09 parametrization. In addition the cross section for the process *γγ*→*e* ^+^ *e* ^−^ has been measured and found to be in agreement with models implementing QED at leading order.

## Introduction

The strong electromagnetic fields generated by heavy ions at the LHC provide an opportunity to study photonuclear interactions in ultra-peripheral collisions (UPC), where the impact parameter may be several tens of femtometres and no hadronic interactions occur. The photon flux is proportional to the square of the nucleus charge, so the photon flux in lead beams is enhanced by nearly four orders of magnitude compared to proton beams. The strong photon flux leads to large cross sections for a variety of photonuclear and two-photon interactions. The physics of ultra-peripheral collisions is described in Refs. [1, 2]. Exclusive vector meson photoproduction, where a vector meson is produced in an event with no other final state particles, is of particular interest, since it provides a measure of the nuclear gluon distribution at low Bjorken-*x*.

Exclusive production of charmonium in photon–proton interactions at HERA, *γ*+p→J/*ψ*(*ψ*′)+p, has been successfully modelled in perturbative QCD in terms of the exchange of two gluons with no net-colour transfer [3]. Exclusive vector meson production at mid-rapidity in heavy-ion collisions has previously been studied at RHIC [4, 5]. The exclusive photoproduction can be either coherent, where the photon couples coherently to almost all the nucleons, or incoherent, where the photon couples to a single nucleon. Coherent production is characterized by low transverse momentum of vector mesons (〈*p*_T_〉≈60 MeV/*c*) where the nucleus normally does not break up by the J/*ψ* production. However, the exchange of additional photons may lead to the nucleus break-up, estimated by the simulation models at the level of 20–30 % of the events. Incoherent production, corresponding to quasi-elastic scattering off a single nucleon, is characterized by a somewhat higher transverse momentum (〈*p*_T_〉≈500 MeV/*c*). In this case the nucleus interacting with the photon breaks up, but, apart from single nucleons or nuclear fragments in the very forward region, no other particles are produced.

Recently the ALICE Collaboration published the first results on the photoproduction of J/*ψ* in ultra-peripheral Pb–Pb collisions at the LHC [6]. This first measurement was performed in the rapidity region −3.6<*y*<−2.6 and allows us to constrain the nuclear gluon distribution at Bjorken-*x*≈10^−2^. In this paper, results from the ALICE experiment on exclusive photoproduction of J/*ψ* and *ψ*′ mesons at mid-rapidity in ultra-peripheral Pb–Pb collisions at $\sqrt{s_{\mathrm{NN}}} = 2.76~\mathrm{TeV}$ are presented. The measurement at mid-rapidity allows the exploration of the region $x = (M_{ \mathrm {J}/\psi }/\sqrt{s_{\mathrm{NN}}})\exp(\pm y)\approx10^{-3}$, where at present the uncertainty in the nuclear gluon shadowing distribution is rather large [7]. This analysis is focused both on coherently and incoherently produced J/*ψ* mesons. The measured cross section is compared to model predictions [8–13].

Two-photon production of di-lepton pairs in heavy-ion interactions is also of great interest, as it probes Quantum Electrodynamics in the regime of strong fields. This process is sensitive to the effect produced by the strong fields of the nuclei. The coupling Z$\sqrt{\alpha}$ is large, so higher-order terms may become important. Predictions exist where these terms are found to lead to a reduction in the cross section by up to 30 % [14, 15]. Other calculations have found agreement with leading-order calculations for muon pairs and electron pairs with invariant masses much larger than two times the electron mass [16]. Measurements at LHC energies can provide useful insights to assess these effects. In this paper we present the study of the *γγ*→*e* ^+^ *e* ^−^ process. The results are compared with predictions by models neglecting higher-order effects discussed above.

## Detector description

The ALICE experiment consists of a central barrel placed in a large solenoid magnet (B=0.5 T), covering the pseudorapidity region |*η*|<0.9, and a muon spectrometer at forward rapidity, covering the range −4.0<*η*<−2.5 [17]. In this analysis the following detectors of the central barrel have been used. The Silicon Pixel Detector (SPD) makes up the two innermost layers of the ALICE Inner Tracking System (ITS), covering extended pseudorapidity ranges |*η*|<2 and |*η*|<1.4, for the inner (radius 3.9 cm) and outer (average radius 7.6 cm) layers, respectively. It is a fine granularity detector, having about 10^7^ pixels, and can be used for triggering purposes. The Time Projection Chamber (TPC) is used for tracking and for particle identification. A 100 kV central electrode separates the two drift volumes, providing an electric field for electron drift, and the two end-plates, at |*z*|=250 cm, are instrumented with Multi-Wire-Proportional-Chambers (MWPCs) with 560 000 readout pads, allowing high precision track measurements in the transverse plane. The *z* coordinate is given by the drift time in the TPC electric field. The TPC acceptance covers the pseudorapidity region |*η*|<0.9. Ionization measurements made along track clusters are used for particle identification [18]. Beyond the TPC, the Time-of-Flight detector (TOF) is a large cylindrical barrel of Multigap Resistive Plate Chambers (MRPCs) with about 150 000 readout channels, giving very high precision timing for tracks traversing it. Its pseudorapidity coverage matches that of the TPC. Used in combination with the tracking system, the TOF detector can be used for charged particle identification up to about 2.5 GeV/*c* (pions and kaons) and 4 GeV/*c* (protons). Still further out from the interaction region, the Electromagnetic Calorimeter (EMCAL) is a Pb-scintillator sampling calorimeter at a distance of ≈4.5 metres from the beam line, covering an opening acceptance in the range |*η*|≤0.7 and *Δϕ*=100^∘^ in azimuth. It has 20.1 radiation lengths and consists of 11 520 towers.

The analysis presented below also makes use of two forward detectors. The VZERO counters consist of two arrays of 32 scintillator tiles each, covering the range 2.8<*η*<5.1 (VZERO-A, on the opposite side of the muon arm) and −3.7<*η*<−1.7 (VZERO-C, on the same side as the muon arm) and positioned respectively at *z*=340 cm and *z*=−90 cm from the interaction point. The Forward Multiplicity Detector (FMD) consists of Si-strip sensors with a total of 51 240 active detection elements, arranged in five rings perpendicular to the beam direction, covering the pseudorapidity ranges −3.4<*η*<−1.7 (FMD-3) and 1.7<*η*<5.1 (FMD-1 and FMD-2), a similar coverage to that of the VZERO detector. Finally, two sets of hadronic Zero Degree Calorimeters (ZDC) are located at 116 m on either side of the interaction point. The ZDCs detect neutrons emitted in the very forward region (|*η*|>8.7), such as neutrons produced by electromagnetic dissociation [19] (see Sect. 3).

## Data analysis

### Event selection

The present analysis is based on a sample of events collected during the 2011 Pb–Pb data-taking, selected with a dedicated barrel ultra-peripheral collision trigger (BUPC), set up to select events containing two tracks in an otherwise empty detector. Events from two-photon production (*γγ*→*μ* ^+^ *μ* ^−^,*e* ^+^ *e* ^−^) or from photonuclear vector meson production are selected by this trigger with the following characteristics:

(i) at least two hits in the SPD detector;
(ii) a number of fired pad-OR (*N* ^on^) in the TOF detector [20] in the range 2≤*N* ^on^≤6, with at least two of them with a difference in azimuth, *Δϕ*, in the range 150^∘^≤*Δϕ*≤180^∘^;
(iii) no hits in the VZERO-A and no hits in the VZERO-C detectors.

A total of about 6.5×10^6^ events were selected by the BUPC trigger.

The integrated luminosity was measured using a trigger for the most central hadronic Pb–Pb collisions. The cross section for this process was obtained with a van der Meer scan [21], giving a cross section $\sigma= 4.10^{+0.22}_{-0.13}$(sys) b [22]. This gives an integrated luminosity for the BUPC trigger sample, corrected for trigger live time, of . An alternative method based on using neutrons detected in the two ZDCs was also used. The ZDC trigger condition required a signal in at least one of the two calorimeters, thus selecting single electromagnetic dissociation (EMD) as well as hadronic interactions. The cross section for this trigger was also measured with a van der Meer scan, giving a cross section $\sigma= 371.4\pm 0.6(\mathrm{sta})\pm^{24}_{19}$(sys) b [19]. The integrated luminosity obtained for the BUPC by this method is consistent with the one quoted above within 3 %.

**Fig. 1:**
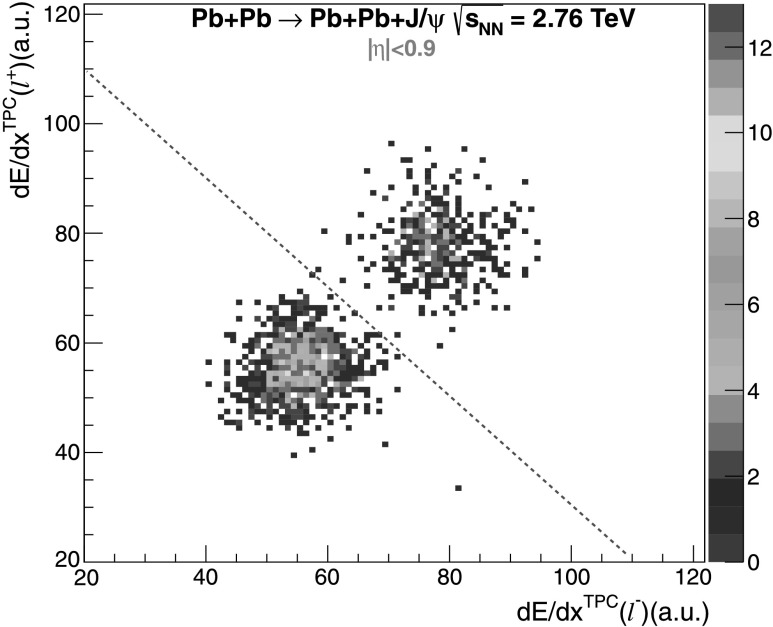
d*E*/d*x* of the positive lepton versus the negative one, as measured by the TPC for J/*ψ* candidates in the ultra-peripheral Pb–Pb collisions at $\sqrt{s_{\mathrm{NN}} } = 2.76~\mathrm{TeV}$ in the invariant mass range 2.8<*M*_inv_<3.2 GeV/*c* ^2^ and −0.9<*η*<0.9. Muon pairs and electron pairs are clearly separated, with the latter showing higher d*E*/d*x* values
(vii) the two tracks have same or opposite charges, depending on the analysis;
(viii) invariant mass 2.2<*M*_inv_<6 GeV/*c* ^2^.

The following selection criteria were applied in the data analysis:

(i) a number of reconstructed tracks 1≤*N*_TRK_≤10, where a track is defined with loose criteria: more than 50 % of findable clusters in the TPC fiducial volume and at least 20 TPC clusters, matching with those found in the ITS;
(ii) a reconstructed primary vertex;
(iii) only two good tracks passing tighter quality cuts: at least 70 TPC clusters, at least 1 SPD cluster and rejection of tracks with a kink. Moreover the tracks extrapolated to the reconstructed vertex should have a distance of closest approach (DCA) in the longitudinal beam direction DCA_*L*_≤2 cm, and $\mathrm{DCA}_{T}\leq0.0182+0.0350/p_{\mathrm{T}}^{1.01}~\mathrm{cm}$ in the plane orthogonal to the beam direction, where *p*_T_ is in (GeV/*c*);
(iv) at least one of the two good tracks selected in (iii) with *p*_T_≥1 GeV/*c*; this cut reduces the background while it does not affect the genuine leptons from J/*ψ* decay;
(v) the VZERO trigger required no signal within a time window of 25 ns around the collision time in any of the scintillator tiles of both VZERO-A and VZERO-C. The time width of the trigger windows are limited by the design of the VZERO front-end electronics which is operated at the frequency of the LHC clock, i.e. 40 MHz. In the offline analysis the event selection criteria consisted in an absence of a reconstructed signal in any of the VZERO scintillator tiles. The time windows in the offline analysis are enlarged to 40 ns and 60 ns around the collision time in VZERO-A and VZERO-C, respectively, and were chosen in order to maximize the vetoing efficiency;
(vi) the d*E*/d*x* for the two tracks is compatible with that of electrons or muons; Fig. 1 shows the TPC d*E*/d*x* of the positive lepton candidate as a function of the d*E*/d*x* of the negative lepton candidate, for J/*ψ* candidates in the invariant mass range 2.8<*M*_inv_<3.2 GeV/*c* ^2^. It is worth noting that the TPC resolution does not allow to distinguish between muons and charged pions;

The analysis of the *γγ* events is discussed in Sect. 5. In the remaining of this section we will focus on J/*ψ* analysis. The effect of the cuts on the statistics is listed in Table 1. In addition to the requirements (i) to (viii), a first sample enriched with coherent events was selected by applying a cut *p*_T_<200 MeV/*c* for di-muons (*p*_T_<300 MeV/*c* for di-electrons). Photoproduction of vector mesons can occur in interactions where additional photons are exchanged [23]. These additional photons can lead to break-up of one or both nuclei. Since the energies of these photons are low, only a few neutrons are emitted when the nuclei break up. The exact upper limit on the number of emitted neutrons is not known, but in this analysis a cut on the neutron ZDC signal corresponding to less than 6 neutrons on each side has been applied. This cut reduces the statistics by 2.5 %, which is considered as a source of systematic error ${}^{+2.5~\%}_{-0~\%}$. After applying all of these selections, 746 di-electron and 1301 di-muon coherent lepton-pair candidates remain. A second sample was enriched with incoherent events by applying a cut *p*_T_>200 MeV/*c* on di-muons (*p*_T_>300 MeV/*c* on di-electrons), giving 278 electron and 1748 muon incoherent event candidates.

**Table 1:** Summary of the applied data cuts (see text)

Selection	Number of remaining events
Triggered events	6,507,692
1≤*N*_TRK_≤10	2,311,056
Primary vertex	1,972,231
Two reconstructed tracks	436,720
$\max(p_{\mathrm{T}}^{1},p_{\mathrm{T}}^{2})>1~\mathrm{GeV}/c$	46,324
VZERO offline	46,183
d*E*/d*x* consistent with electron (muon)	45,518
Opposite sign tracks	31,529
2.2<*M*_inv_<6.0 GeV/*c* ^2^	4,542

As described in reference [6], during the 2011 Pb–Pb run the VZERO detector was optimized for the selection of hadronic Pb–Pb collisions, with a threshold corresponding to an energy deposit above that from a single minimum ionizing particle. Since the VZERO was used as a veto in the BUPC trigger, this setting could lead to an inefficiency in background rejection. In about 30 % of the 2011 BUPC data taking sample, the FMDs were read out too. Since these detectors cover a pseudorapidity interval similar to that of the VZEROs, we have used, offline, their information to check for possible inefficiencies in the VZERO data. As expected, we found no hits in the FMD detector for the selected BUPC events, confirming that the VZERO inefficiency is very small.

A test on the electron and muon separation was applied to those tracks crossing the EMCAL. For each track we evaluated the ratio *E*/*p* between the energy released in the EMCAL and the reconstructed momentum; electrons lose their total energy in the shower generated in the EMCAL and for these a value *E*/*p*≈1 is measured. Minimum ionizing particles lose only a small fraction of their energy in the EMCAL; in this case the measured *E*/*p* peaks in the region 0.1–0.2, in good agreement with the expectation. The *e*/*μ* separation was obtained by using two methods: (a) a sharp cut on Fig. 1, where all the particles beyond the dotted line are considered as electrons, and (b) using the average of the electron (muon) d*E*/d*x* and considering as electrons (muons) the particles within 3 sigma. The difference between the two methods was used as an estimate of the systematic error (see Table 2).

**Table 2:** Summary of the contributions to the systematic error for the J/*ψ* and *γγ* cross section measurement for electrons (muons). The error for the J/*ψ* signal extraction includes the systematic error in the fit of the invariant mass spectrum and the systematic errors on *f*_*D*_ and *f*_*I*_(*f*_*C*_), as described in the text

Source	Coherent	Incoherent	*γγ* (low)	*γγ* (high)
Luminosity	${}^{+5~\%}_{-3~\%}$	${}^{+5~\%}_{-3~\%}$	${}^{+5~\%}_{-3~\%}$	${}^{+5~\%}_{-3~\%} $
Trigger dead time	±2.5 %	±2.5 %	±2.5 %	±2.5 %
Signal extraction	${}^{+7~\%}_{-6~\%}({}^{+6~\%}_{-5~\%})$	${}^{+26.5~\%}_{-12.5~\%}({}^{+9~\%}_{-8~\%})$	±1 %	±4 %
Trigger efficiency	${}^{+3.8~\%}_{-9.0~\%}$	${}^{+3.8~\%}_{-9.0~\%}$	${}^{+3.8~\%}_{-9.0~\%}$	${}^{+3.8~\%}_{-9.0~\%}$
(Acc×*ε*)	±2.5 (±1) %	±6.5 (±3.5) %	±0.3 %	±0.5 %
*γγ*→*e* ^+^ *e* ^−^ background	${}^{+4~\%}_{-0~\%}$	${}^{+4~\%}_{-0~\%}$	${}^{+4~\%}_{-0~\%}$	${}^{+4~\%}_{-0~\%}$
*e*/*μ* separation	±2 %	±2 %	±1.7 %	±4 %
Branching ratio	±1 %	±1 %	–	–
Neutron number cut	${}^{+2.5~\%}_{-0~\%}$	–	–	–
Hadronic J/*ψ*	–	${}^{+0~\%}_{-5~\%}$ (${}^{+0~\%}_{-3~\%}$)	–	–
Total	${}^{+14.0~\%}_{-9.6~\%}({}^{+13.4~\%}_{-8.8~\%})$	${}^{+29.4~\%}_{-16.6~\%}({}^{+14.5~\%}_{-11.7~\%})$	${}^{+10.8~\%}_{-7.0~\%}$	${}^{+12.0~\%}_{-8.8~\%}$

### Acceptance and efficiency correction

The acceptance and efficiency of J/*ψ* reconstruction were calculated using a large sample of coherent and incoherent J/*ψ* events generated by STARLIGHT [24] and folded with the detector Monte Carlo simulation. STARLIGHT simulates photonuclear and two-photon interactions at hadron colliders. The simulations for exclusive vector meson production and two-photon interactions are based on the models in [9] and [25], respectively.

A separate simulation was performed for each run, in order to take into account the slight variations in run conditions during the data taking. The product of the acceptance and efficiency corrections (Acc×*ε*)_J/*ψ*_ was calculated as the ratio of the number of the simulated events that satisfy all selections in Table 1 to the number of generated events with the J/*ψ* in the rapidity interval −0.9<*y*<0.9. In addition, the reconstructed transverse momentum is required to be *p*_T_<200 MeV/*c* (*p*_T_>200 MeV/*c*) for di-muons and *p*_T_<300 MeV/*c* (*p*_T_>300 MeV/*c*) for di-electrons in the coherent (incoherent) sample.

The average values for the combined acceptance and efficiency for J/*ψ*→*e* ^+^ *e* ^−^(*μ* ^+^ *μ* ^−^) were found to be 2.71 (4.57) % and 1.8 (3.19) % for coherent and incoherent J/*ψ*, respectively. The STARLIGHT model predicts a dependence of the J/*ψ* cross section on the rapidity, giving a ≈10 % variation over the rapidity range *y*=±0.9. In order to evaluate the systematic error on the acceptance coming from the generator choice, we used a flat dependence of d*σ*_J/*ψ*_/d*y* in the interval −0.9<*y*<0.9, as predicted by other models (see Fig. 6). The relative differences in (Acc×*ε*) between the methods were 2.5 (1.0) % for coherent electrons (muons), and 6.5 (3.5) % for incoherent electrons (muons), and are taken into account in the systematic error calculation. Transverse polarization is expected from helicity conservation for a quasi-real photon. It is therefore assumed in these calculations that the J/*ψ* is transversely polarized, as found by previous experiments [26, 27]. The trigger efficiency was measured relying on a data sample collected in a dedicated run triggered by the ZDCs only. We selected events with a topology having the BUPC conditions, given at the beginning of Sect. 3.1. The resulting trigger efficiency was compared with that obtained by the Monte Carlo simulation, showing agreement within ${}^{+3.8~\%}_{-9.0~\%}$.

### Analysis of invariant mass spectrum

**Fig. 2:**
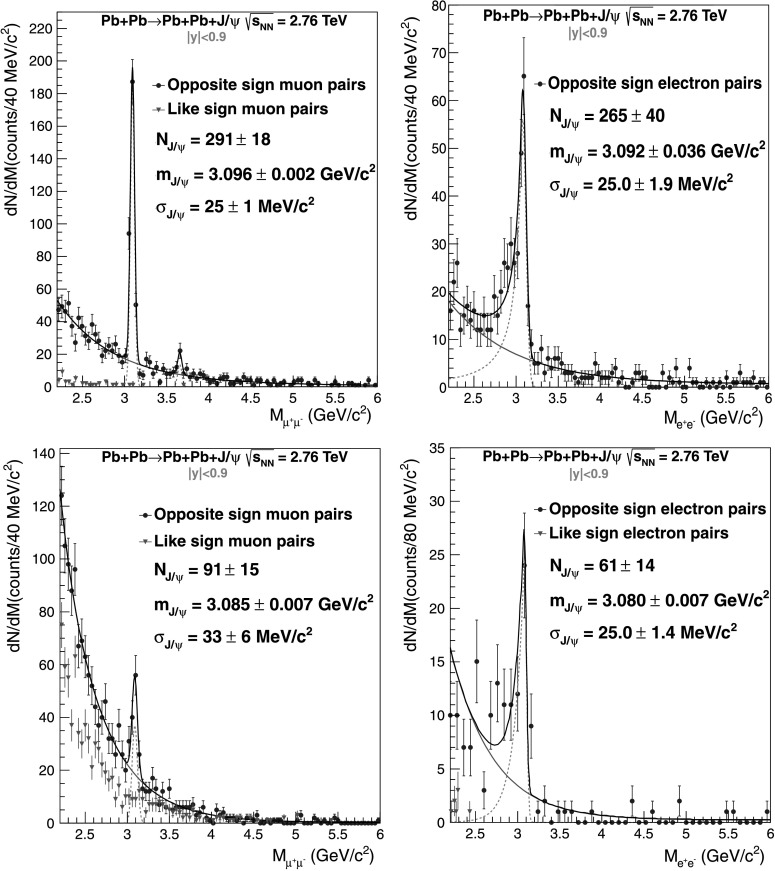
Invariant mass distributions for ultra-peripheral Pb–Pb collisions at $\sqrt{s_{\mathrm{NN}}} = 2.76~\mathrm{TeV}$ and −0.9<*y*<0.9 for events satisfying the event selection in Table 1, in the invariant mass range 2.2<*M*_inv_<6 GeV/*c* ^2^. Coherent enriched sample (*top*) and incoherent enriched sample (*bottom*) for muons (*left*) and electrons (*right*). *Blue* (*red*) *circles* (*triangles*) are opposite-sign (like-sign) pairs. For like-sign pair the penultimate cut in Table 1 is replaced by the request of a same-sign pair. No LS events were found for coherent di-electron events

Figure 2 shows the invariant mass distribution for 2.2<*M*_inv_<6.0 GeV/*c* ^2^ for opposite-sign (OS) and like-sign (LS) electron and muon pairs. A J/*ψ* peak is clearly visible in the four spectra, on top of a continuum coming from *γγ*→*e* ^+^ *e* ^−^(*μ* ^+^ *μ* ^−^) for the coherent enriched sample. The continuum for the incoherent enriched sample for the muon channel (bottom, left) is likely to come from misidentified *π* ^+^ *π* ^−^ pairs. To extract the J/*ψ* yield, the number of OS events in the interval 2.2<*M*_inv_<3.2 GeV/*c* ^2^ for electrons and 3.0<*M*_inv_<3.2 GeV/*c* ^2^ for muons were considered. In the mass intervals quoted above, 0 (3) LS electron(muon)-pairs were found for coherent enriched events, while 8 (53) LS pairs were found for incoherent enriched events. The corresponding number of OS pairs was 514 (365) for coherent enriched sample and 143 (178) for incoherent enriched events. The J/*ψ* yield was obtained by fitting the di-lepton invariant mass spectrum with an exponential function to describe the underlying continuum, and a Crystal Ball function [28] to extract the J/*ψ* signal. The Crystal Ball function tail parameters (*α*_CB_ and *n*) were left free for the coherent enriched sample, giving a good agreement with those obtained by fitting the simulated data, and were fixed to values obtained from simulations for the incoherent enriched one. The background found in the incoherent sample was taken into account into the fit by using a fifth-order polynomial in addition to the Crystal Ball and an exponential function. This contribution was normalized according to the experimental LS pair spectrum (Fig. 2).

## The J/*ψ* cross section

### Coherent J/*ψ* cross section

The yield obtained for the coherent enriched sample (Fig. 2 top) is $N_{\rm yield} = 265 \pm40\rm{(sta)}\pm12\rm{(sys)}$ for the J/*ψ*→*e* ^+^ *e* ^−^ channel and $N_{\rm yield} = 291 \pm18\rm{(sta)}\pm4\rm{(sys)}$ for the J/*ψ*→*μ* ^+^ *μ* ^−^ channel. The systematic error on the yield is obtained by varying the bin size and by replacing the exponential with a polynomial to fit the *γγ* process. In addition, the Crystal Ball function parameters are obtained by fitting a simulated sample made of J/*ψ* and *γγ* event cocktail and then used to fit the coherent enriched data sample too. The difference in the yield obtained with the two Crystal Ball fit procedures is included in the systematic error. As a result we obtain a ${}^{+7~\%}_{-6~\%}$ and ${}^{+6~\%}_{-5~\%}$ systematic error on the signal extraction for coherent electrons and muons, respectively. For the coherent enriched sample, the central mass (width calculated from the standard deviation) value from the fit is 3.092±0.036 GeV/*c* ^2^ (25.0±1.9 MeV/*c* ^2^) for the electron channel and 3.096±0.002 GeV/*c* ^2^ (25±1.1 MeV/*c* ^2^) for the muon channel, in good agreement with the known value of the J/*ψ* mass and compatible with the absolute calibration accuracy of the barrel. For the incoherent sample, the central mass (width calculated from the standard deviation) value from the fit is 3.080±0.007 GeV/*c* ^2^ (25±1.4 MeV/*c* ^2^) for the electron channel and 3.085±0.007 GeV/*c* ^2^ (33±6 MeV/*c* ^2^) for the muon channel. The exponential slope parameter, *λ*_*γγ*_, of the continuum for the coherent enriched sample is computed at 2.2<*M*_inv_<2.6 GeV/*c* ^2^ (low) and 3.7<*M*_inv_<10 GeV/*c* ^2^ (high) for electrons with −0.9<*η*_1,2_<0.9, giving −1.55±0.88 GeV^−1^ *c* ^2^ and −0.73±0.18 GeV^−1^ *c* ^2^, in good agreement with the corresponding Monte Carlo expectation, −1.07±0.16 GeV^−1^ *c* ^2^ and −0.81±0.01 GeV^−1^ *c* ^2^, respectively. This is an additional indication that there is no important background in the invariant mass and *p*_T_ range considered.

**Fig. 3:**
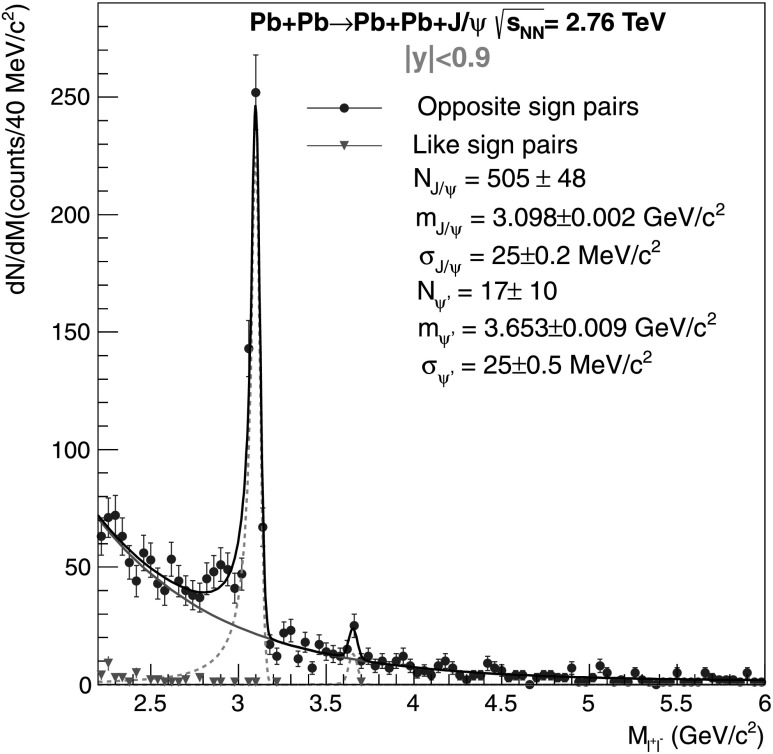
Invariant mass distribution for ultra-peripheral Pb–Pb collisions at $\sqrt{s_{\mathrm{NN}} } = 2.76~\mathrm{TeV}$ at −0.9<*y*<0.9 for events satisfying the event selection in Table 1, in the invariant mass interval 2.2<*M*_inv_<6 GeV/*c* ^2^. Coherent di-electron and di-muon candidates are summed together. For like-sign pair the penultimate cut in Table 1 is replaced by the request of a same-sign pair

Exclusive photoproduction of *ψ*′, followed by the *ψ*′→J/*ψ*+anything decay, can be a background for this analysis when particles produced in addition to the J/*ψ* are undetected. The fraction *f*_*D*_ of coherent J/*ψ* mesons coming from the decay *ψ*′→J/*ψ*+anything, was estimated following the same prescription used in [6], with the theoretical estimates for *f*_*D*_ ranging from 4.4 % to 11.8 % for electrons and 4.3 % to 14.7 % for muons. Alternatively, the ratio of coherent yields for *ψ*′ to J/*ψ* can be extracted from the real data. Owing to the limited statistics, we combine the electron and muon channels to obtain *N*_*ψ*′_=17±10 and *N*_*ψ*_=505±48 (see Fig. 3). The fraction *f*_*D*_, for a given J/*ψ* polarization *P*, can be written as:

1 $$\begin{aligned} f_{D}^{P} =&\frac { N_{\psi'}\cdot (\mathrm{ Acc}\times\varepsilon)_{\psi'\rightarrow \mathrm {J}/\psi }^{P} }{ (\mathrm{Acc}\times\varepsilon)_{\psi'\rightarrow{l}^{+}l^{-}} } \\ &{}\times \frac{ \mathit{BR}(\psi' \rightarrow \mathrm {J}/\psi +\mathrm{anything}) }{ \mathit{BR}(\psi' \rightarrow l^{+}l^{-}) } \\ &{}\times \frac {\mathit{BR}( \mathrm {J}/\psi \rightarrow l^{+}l^{-})}{ N_{ \mathrm {J}/\psi }}, \end{aligned}$$

where $(\mathrm{ Acc}\times\varepsilon)_{\psi'\rightarrow \mathrm {J}/\psi }^{P}$ ranges from 2 % to 3 % for electrons and from 3.4 % to 4.6 % for muons, depending on the J/*ψ* polarization. The $(\mathrm{Acc}\times\varepsilon)_{\psi'\rightarrow{l}^{+}l^{-}}^{P}$ ranges from 3.3 % to 4.5 % for electrons and muons, respectively. The acceptance corrections are polarization dependent and give $f_{D}^{P}$ ranging from 15±9 % for longitudinal polarization to 11±6.5 % for transverse polarization.

**Fig. 4:**
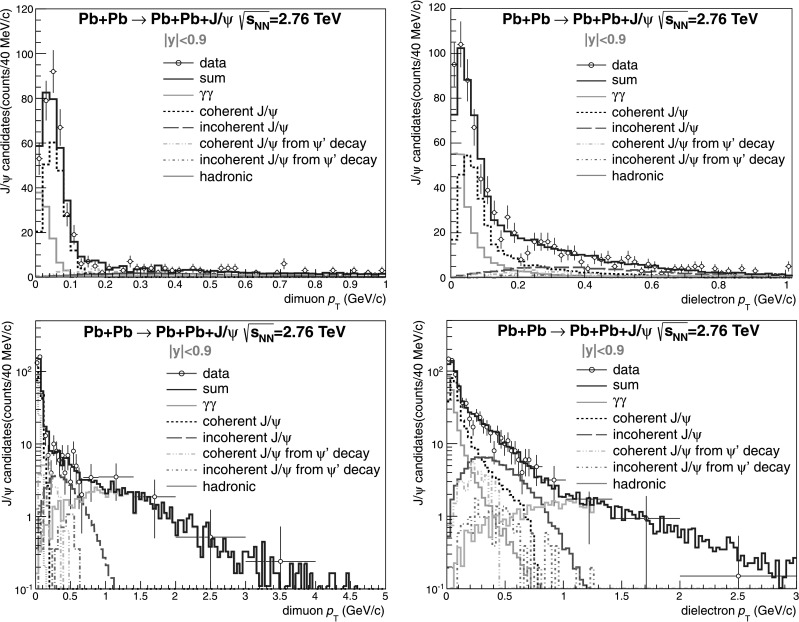
Di-muon (*left*) and di-electron (*right*) *p*_T_ distribution for ultra-peripheral Pb–Pb collisions at $\sqrt{s_{\mathrm{NN}} } = 2.76~\mathrm{TeV}$ and −0.9<*y*<0.9 for events satisfying the event selection in the invariant mass interval 3.0<*M*_inv_<3.2 GeV/*c* ^2^ and 2.2<*M*_inv_<3.2 GeV/*c* ^2^, respectively, with the *p*_T_-range extended to *p*_T_<1 GeV/*c* (*top*) and to *p*_T_<5 GeV/*c* (*bottom*). The data points are fitted summing six different Monte Carlo templates: coherent J/*ψ* production (*black*), incoherent J/*ψ* production (*red*), J/*ψ*s from coherent *ψ*′ decay (*light blue*), J/*ψ*s from incoherent *ψ*′ decay (*violet*), *γγ* (*green*), and J/*ψ* produced in peripheral hadronic collisions (*grey*). The *solid histogram* (*blue*) is the sum (Color figure online)

In what follows, we use the central value of theoretical and experimental estimates, and take the others as upper and lower limits, i.e. $f_{D}=0.10^{+0.05}_{-0.06}$. The di-electron (di-muon) *p*_T_ distribution, integrated over 2.2<*M*_inv_<3.2 GeV/*c* ^2^, (3.0<*M*_inv_<3.2 GeV/*c* ^2^) is shown in Fig. 4 right (left). The clear peak at low *p*_T_ is mainly due to coherent interactions, while the tail extending out to 1 GeV/*c* comes from incoherent production. To estimate the fraction (*f*_*I*_) of incoherent over coherent events in the low *p*_T_ region (*p*_T_<300 MeV/*c* for di-electrons, *p*_T_<200 MeV/*c* for di-muons), the ratio *σ*_inc_/*σ*_coh_, weighted by the detector acceptance and efficiency for the two processes, was calculated, giving *f*_*I*_=0.13 (0.06) for di-electrons (di-muons) when *σ*_inc_/*σ*_coh_ was taken from STARLIGHT, and *f*_*I*_=0.05 (0.03) when the model in [8] was used with leading twist contribution. For higher twist contributions the above model gives *f*_*I*_=0.07 (0.03). An alternative method to extract an upper limit of *f*_*I*_ from the data was carried out by fitting the measured *p*_T_ distribution. Six different functions were used to describe the *p*_T_ spectrum:

(i) coherent J/*ψ* photoproduction;
(ii) incoherent J/*ψ* photoproduction;
(iii) J/*ψ* from coherent *ψ*′ decay;
(iv) J/*ψ* from incoherent *ψ*′ decay;
(v) two-photon production of continuum pairs;
(vi) J/*ψ* produced in peripheral hadronic collisions.

The shapes for the first five fitting functions (Monte Carlo templates) were provided by STARLIGHT events folded with the detector simulation, while the last one is extracted from data at higher centralities [29]. The relative normalization was left free for coherent and incoherent photoproduction. The contribution from the *ψ*′ was constrained from the estimate above ($f_{D}=0.10^{+0.05}_{-0.06}$), and the two-photon contribution was determined from the fit to the continuum in Fig. 2. The hadronic J/*ψ* were constrained by the fit to the region *p*_T_>1.1 GeV/*c*, where the ultra-peripheral J/*ψ* contribution is negligible. As a result of the fit, we obtain *f*_*I*_=0.044±0.014 for di-muons and *f*_*I*_=0.15±0.02 for di-electrons. Since these values are compatible, within the errors, with the theoretical expectations (for both models in the case of di-muons and for STARLIGHT for the di-electrons), they are used in the calculations. The fit reproduces properly the experimental data *p*_T_ spectrum, clarifying the origin of the high *p*_T_ J/*ψ*s pointed out in the PHENIX paper [5]. Finally, the total number of coherent J/*ψ*s is calculated from the yield extracted from the fit to the invariant mass distribution by

2 $$ N^{\mathrm{coh}}_{ \mathrm {J}/\psi } = \frac{N_{\rm yield}}{1 + f_I + f_D} , $$

resulting in $N^{\mathrm{coh}}_{ \mathrm {J}/\psi }(\mu^{+}\mu^{-}) = 255\pm 16(\mathrm{sta}) ^{+14}_{-13} (\mathrm{sys}) $ and $N^{\mathrm{coh}}_{ \mathrm {J}/\psi }(e^{+}e^{-}) =212 \pm32(\mathrm{sta}) ^{+14}_{-13} (\mathrm{sys})$, respectively. The coherent J/*ψ* differential cross section is given by

3 $$ \frac{\mathrm{d}\sigma_{ \mathrm {J}/\psi }^{\mathrm{coh}}}{\mathrm{d}y} = \frac{N_{ \mathrm {J}/\psi }^{\mathrm{coh}}}{ (\mathrm {Acc}\times \varepsilon )_{\mathrm {J}/\psi } \cdot \mathit{BR}( \mathrm {J}/\psi \rightarrow l^{+}l^{-}) \cdot{\mathcal{L}_{\mathrm{int}}} \cdot \varDelta y } , $$

where $N^{\mathrm{coh}}_{ \mathrm {J}/\psi }$ is the number of J/*ψ* candidates from Eq. (2) and (Acc×*ε*)_J/*ψ*_ corresponds to the acceptance and efficiency as discussed above. *BR*(J/*ψ*→*l* ^+^ *l* ^−^) is the branching ratio for J/*ψ* decay into leptons [30], $\varDelta \it{y} =1.8$ the rapidity interval bin size, and $\mathcal {L}_{\mathrm{int}}$ the total integrated luminosity. As a result we obtain $\mathrm{d}\sigma_{\mathrm{J}/\psi}^{\mathrm{coh}} /\mathrm{d}y = 2.27 \pm 0.14(\mathrm{sta})^{+0.30}_{-0.20}(\mathrm{sys})~\mathrm{mb}$ for the di-muon channel and $\mathrm{d}\sigma_{\mathrm{J}/\psi}^{\mathrm{coh}} / \mathrm{d}y = 3.19\pm 0.50(\mathrm{sta})^{+0.45}_{-0.31}(\mathrm{sys})~\mathrm{mb}$ for the electron one. Since the di-electron and di-muon data are statistically separated samples, they can be combined; their weighted average gives $\mathrm{d}\sigma_{\mathrm{J}/\psi}^{\mathrm{coh}} /\mathrm{d}y = 2.38^{+0.34}_{-0.24} (\mathrm{sta+sys} )~\mathrm{mb}$.

In addition, the fraction *F*_*n*_ of coherent events with no neutron emission was estimated by STARLIGHT to be *F*_*n*_=0.68, while the model [8] predicts *F*_*n*_=0.76. Events with neutron emission can be efficiently tagged in ALICE by the ZDC calorimeters, taking advantage of their high efficiency (>98 %). By fitting the di-electron (di-muon) invariant mass spectrum for events with and without neutron emission and with *p*_T_<300 MeV/*c* (*p*_T_<200 MeV/*c*), we obtain a fraction $0.70\pm 0.05\mathrm{(sta)}$ in good agreement with the above estimates.

### Incoherent J/*ψ* cross section

The incoherent cross sections are obtained in a similar way. For the incoherent enriched sample the obtained yield is (Fig. 2 bottom), $N_{\rm yield} =61 \pm14\rm{(sta)}^{+16}_{-7}\rm{(sys)}$ for the J/*ψ*→*e* ^+^ *e* ^−^ channel and $N_{\rm yield} = 91 \pm15\rm{(sta)}^{+7}_{-5}\rm{(sys)}$ for the J/*ψ*→*μ* ^+^ *μ* ^−^ channel. Here *f*_*D*_ represents the fraction of incoherent J/*ψ* mesons coming from the decay *ψ*′→J/*ψ*+anything, and was obtained only from formula (1), since the limited statistics did not allow the extraction of the *ψ*′ yield from the data. The predictions for incoherent *f*_*D*_ are calculated using both STARLIGHT and the model [8]. As a result we obtain a *f*_*D*_ value ranging from 3.9 % to 15.1 % for muons and from 3.8 % to 18.1 % for electrons. By using the average we obtain *f*_*D*_=(9.5±5.5)% for muons and *f*_*D*_=(11±7)% for electrons. Using STARLIGHT, *f*_*C*_ (the fraction of coherent J/*ψ* contaminating the incoherent sample), corrected by the acceptance and the efficiency, is found to be *f*_*C*_=0.5 for electrons and *f*_*C*_=0.02 for muons. By fitting the measured *p*_T_ distribution (Fig. 4) we extract *f*_*C*_=(0.47±0.09) for electrons, while *f*_*C*_ is *f*_*C*_=(0.03±0.03) for muons. These results are compatible with those from the models and will be used in the following. By applying the ratio 1/(1+*f*_*D*_+*f*_*C*_) to the $N_{\rm yield}$, the total number of incoherent muon events is $N^{\mathrm{inc}}_{ \mathrm {J}/\psi }(\mu^{+}\mu^{-})~=81\pm13(\mathrm {sta})^{+8}_{-6} (\mathrm{sys}) $, corresponding to $\mathrm{d}\sigma_{\mathrm{J}/\psi}^{\mathrm{incoh}} /\mathrm{d}y = 1.03 \pm0.17(\mathrm{sta})^{+0.15}_{-0.12}(\mathrm{sys})~\mathrm{mb}$ for the di-muon channel. For electrons we obtain $N^{\mathrm{inc}}_{ \mathrm {J}/\psi }(e^{+}e^{-}) = 39\pm9(\mathrm {sta})^{+10}_{-5}(\mathrm{sys})$, corresponding to $\mathrm{d}\sigma_{\mathrm{J}/\psi}^{\mathrm{incoh}} /\mathrm{d}y = 0.87\pm 0.20(\mathrm{sta})^{+0.26}_{-0.14}(\mathrm{sys})~\mathrm{mb}$ for the di-electron channel. Since these are statistically separate channels, their weighted average gives $\mathrm{d}\sigma_{\mathrm{J}/\psi}^{\mathrm{incoh}} /\mathrm{d}y = 0.98^{+0.19}_{-0.17} (\mathrm{sta+sys} )~\mathrm{mb}$.

### Background and systematic error estimate

As discussed in [6], a possible loss of events might come from correlated QED pair production, i.e. interactions which produce both a J/*ψ* and a low mass *e* ^+^ *e* ^−^ pair (the latter process has a very large cross section), with one of the electrons hitting the VZERO detector and thus vetoing the event. This effect was studied in [6], with a control data sample where no veto at trigger level was applied. As a result, an upper limit on the inefficiency smaller than 2 % was found. In the forward rapidity trigger only VZERO-A was used as a veto, and therefore we estimate, conservatively, a 4 % systematic error for this study.

Another possible source of systematic error is the radiative decay J/*ψ*→*e* ^+^ *e* ^−^, neglected by the event generator used in this paper. We simulated a J/*ψ*→*e* ^+^ *e* ^−^ sample, where 15 % of the events had a photon in the final state [31]. The Crystal Ball function fit applied to this sample provides fit parameters identical to those of the standard sample, and the (Acc×*ε*) is also not distinguishable from the standard value, so no correction is required in this analysis.

A possible background from hadronic J/*ψ* is found (Fig. 4) to be negligible for *p*_T_ below around 200–300 MeV/*c*, and therefore it is not important for coherent production. For incoherent events this background was evaluated from the *p*_T_ fit described above and gives a contribution (0.043±0.015) for di-electrons and (0.024±0.017) for di-muons. These fractions refer to events in the mass interval 2.2<*M*_inv_<3.2 GeV/*c* ^2^ for di-electrons and 3.0<*M*_inv_<3.2 GeV/*c* ^2^ for di-muons, respectively, and therefore are not necessarily J/*ψ* only. We use these fractions as upper limits to be included in the systematic error, giving a contribution ${}^{+0~\%}_{-5~\%}$ and ${}^{+0~\%}_{-3~\%}$, respectively. The hadronic combinatorial background can be estimated by LS events (see Table 2). It is negligible for coherent events and for incoherent di-electrons. For incoherent di-muons this background, possibly coming from misidentified pion pairs, was taken into account by using a polynomial function in the corresponding fit, as described at the end of Sect. 3.

Another source of background may come from photo-produced J/*ψ* by nuclei with impact parameters *b*<2*R*. According to a simulation, based on a calculation method similar to STARLIGHT, the cross section for this process (usually not included in the event generator) is 1.1 mb and 0.7 mb in the centrality bin (80–90) % and (90–100) %, respectively. The survival probability of the events in these two bins was simulated with 2.2×10^6^ Pb–Pb minimum bias events produced by the HIJING event generator. Assuming the trigger conditions (i, ii, Sect. 3) and the analysis cuts (ii, iv, vi, Sect. 3) to be fully satisfied by di-leptons produced in UPC J/*ψ* decays, we find the fraction of events passing the trigger cut (iii) and the analysis cuts (iii, v) to be 0.06 % and 0.3 % in the two centrality bins. This process therefore gives a negligible contribution to the ultra-peripheral cross section.

## Two-photon cross section

**Fig. 5:**
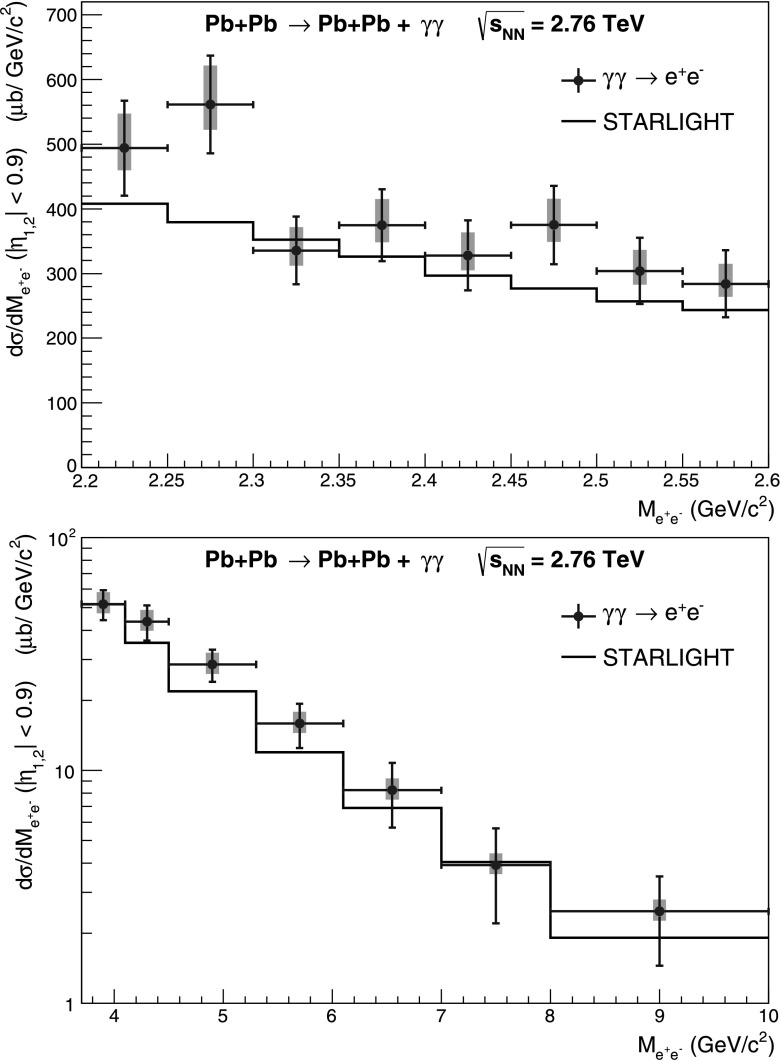
*γγ* →*e* ^+^ *e* ^−^ cross section (*blue circles*) for ultra-peripheral Pb–Pb collisions at $\sqrt{s_{\mathrm{NN}}} = 2.76~\mathrm{TeV}$ at −0.9<*η*<0.9 for events in the invariant mass interval 2.2<*M*_inv_<2.6 GeV/*c* ^2^ (*top*) and 3.7<*M*_inv_<10 GeV/*c* ^2^ interval (*bottom*) compared to STARLIGHT simulation (*black line*). The *blue* (*green*) *bars* show the statistical (systematic) errors, respectively

The STAR Collaboration measured the two-photon cross section with a precision of 22.5 % when adding the statistical and systematic errors in quadrature [32]. This result was slightly larger than the one predicted by STARLIGHT, but within ∼2*σ*. The PHENIX Collaboration has also measured the cross section of two-photon production of di-electron pairs [5]. This measurement, which has an uncertainty of about 30 %, when the statistical and systematic errors are added in quadrature, was found to be in good agreement with STARLIGHT. The cross section for *γγ*→*e* ^+^ *e* ^−^ can be written in a similar way to Eq. (3),

4 $$ \sigma_{\gamma\gamma} = \frac{N_{\gamma\gamma}}{ (\mathrm{Acc}\times\varepsilon)_{\gamma\gamma}\cdot \mathcal{L}_{\mathrm{int}}}, $$

where *N*_*γγ*_ was obtained by fitting the continuum in the invariant mass intervals 2.2<*M*_inv_<2.6 GeV/*c* ^2^ ($N_{\gamma\gamma}^{e^{+} e^{-}}=186\pm13\mathrm{(sta)}\pm4\mathrm{(sys)}$) and 3.7<*M*_inv_<10 GeV/*c* ^2^ ($N_{\gamma\gamma }^{e^{+} e^{-}} =93\pm10\mathrm{(sta)}\pm4\mathrm{(sys)}$), to avoid contamination from the J/*ψ* peak. In this analysis the integrated luminosity used was  and the cut (iv) on the track *p*_T_ was removed. The cross section for the process *γγ*→*μ* ^+^ *μ* ^−^ was not studied due to a possible contamination (although small) from pions in the di-muons sample, suggested by the presence of LS events. The cross section for di-lepton invariant mass was computed between 2.2<*M*_inv_<2.6 GeV/*c* ^2^ and 3.7<*M*_inv_<10 GeV/*c* ^2^, for a di-lepton rapidity in the interval −0.9<*y*<0.9, and requiring −0.9<*η*_1,2_<0.9 for each lepton. The data cuts applied to the Monte Carlo sample are the same as those applied in the analysis described above, resulting in a $(\mathrm{Acc}\times \varepsilon)_{\gamma\gamma}^{e^{+} e^{-}}=5.6~\%$ for 2.2<*M*_inv_<2.6 GeV/*c* ^2^ and $(\mathrm{Acc}\times\varepsilon)_{\gamma\gamma}^{e^{+} e^{-}}=4.73~\%$ for 3.7<*M*_inv_<10 GeV/*c* ^2^. As a result we obtain  for the lower invariant mass interval and  for the higher invariant mass interval, to be compared with *σ*=128 μb and *σ*=77 μb given by STARLIGHT, respectively. In Fig. 5 the invariant mass distributions for 2.2<*M*_inv_<2.6 GeV/*c* ^2^ interval and for 3.7<*M*_inv_<10 GeV/*c* ^2^ are shown. The relevant parameters of the present paper analysis are listed in Table 3.

**Table 3:** Summary of the main experimental results obtained in the J/*ψ* and *γγ* analysis and of the most relevant correction parameters applied

Sample	Coherent enriched J/*ψ*→*μ* ^+^ *μ* ^−^	Coherent enriched J/*ψ*→*e* ^+^ *e* ^−^	Incoherent enriched J/*ψ*→*μ* ^+^ *μ* ^−^	Incoherent enriched J/*ψ*→*e* ^+^ *e* ^−^	*γγ*→*e* ^+^ *e* ^−^ (low)	*γγ*→*e* ^+^ *e* ^−^ (high)
Yield	291±18(sta)	265±40(sta)	91±15(sta)	61±14(sta)	186±13(sta)	93±10(sta)
	± 4(sys)	± 12(sys)	${}^{+7}_{-5}$(sys)	${}^{+16}_{-7}$(sys)	± 12(sys)	± 6(sys)
Mass (GeV/*c* ^2^)	3.096±0.002	3.092±0.036	3.085±0.007	3.080±0.007	–	–
*σ* (MeV/*c* ^2^)	25±1.1	25.0±1.9	33±6	25.0±1.4	–	–
Acc×*ε* (%)	4.57	2.71	3.19	1.8	5.6	4.73
LS pairs	3	0	53	8	0	0
OS pairs	365	514	178	143	186	93
*f*_*D*_	$0.1^{+0.05}_{+ 0.06}$	$0.1^{+0.05}_{+ 0.06}$	0.095±0.055	0.11±0.07	–	–
*f*_*I*_	0.044±0.014	0.15±0.02	–	–	–	–
*f*_*c*_	–	–	0.03±0.03	0.47±0.09	–	–
*λ*_*γγ*_ (GeV^−1^ *c* ^2^)	–	–	–	–	−1.55±0.88	−0.73±0.18

## Discussion

**Fig. 6:**
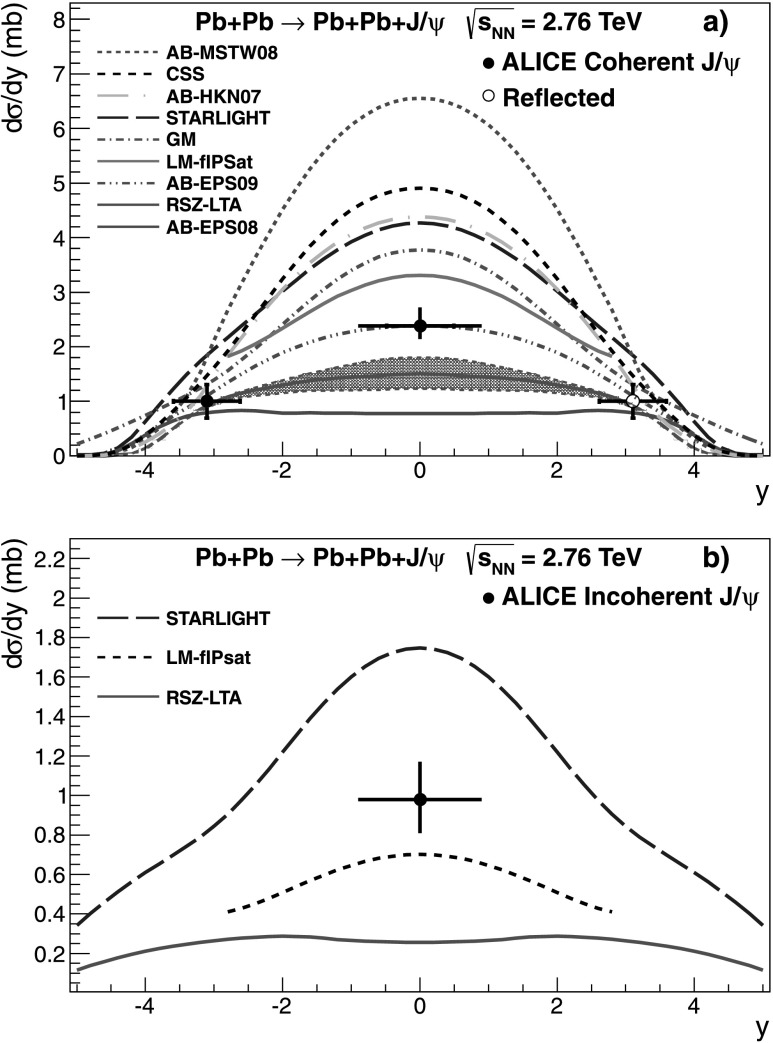
Measured differential cross section of J/*ψ* photoproduction in ultra-peripheral Pb–Pb collisions at $\sqrt{s_{\mathrm{NN}} } = 2.76~\mathrm{TeV}$ at −0.9<*y*<0.9 for coherent (**a**) and incoherent (**b**) events. The error is the quadratic sum of the statistical and systematic errors. The theoretical calculations described in the text are also shown

The cross section of coherent J/*ψ* photoproduction is compared with calculations from six different models [8–13] in Fig. 6(a). The incoherent production cross section is compared with calculations by three different models [8, 9, 13]. These models calculate the photon spectrum in impact parameter space in order to exclude interactions where the nuclei interact hadronically. The differences between the models come mainly from the way the photonuclear interaction is treated. The predictions can be divided into three categories:

(i) those that include no nuclear effects (AB-MSTW08, see below for definition). In this approach, all nucleons contribute to the scattering, and the forward scattering differential cross section, d*σ*/d*t* at *t*=0 (*t* is the momentum transfer from the target nucleus squared), scales with the number of nucleons squared, *A* ^2^;
(ii) models that use a Glauber approach to calculate the number of nucleons contributing to the scattering (STARLIGHT, GM, CSS and LM). The calculated cross section depends on the total J/*ψ*-nucleon cross section and on the nuclear geometry;
(iii) partonic models, where the cross section is proportional to the nuclear gluon distribution squared (AB-EPS08, AB-EPS09, AB-HKN07, and RSZ-LTA).

The rapidity region −0.9<*y*<0.9 considered here corresponds to photon–proton centre-of-mass energies, *W*_*γ*p_, between 59 GeV and 145 GeV. The corresponding range in Bjorken-*x* is between *x*=5×10^−4^ and *x*=3×10^−3^. In this region, a rather strong shadowing is expected, and models based on perturbative QCD predict a lower value for the cross section than models using a Glauber approach to account for the nuclear effect.

The measured cross section, $\mathrm{d}\sigma_{\mathrm{J}/\psi}^{\mathrm{coh}} /\mathrm{d}y = 2.38^{+0.34}_{-0.24} (\mathrm{sta}+\mathrm{sys} )~\mathrm{mb}$ is in very good agreement with the calculation by Adeluyi and Bertulani using the EPS09 nuclear gluon prediction. The GM model, and the other models using a Glauber approach, predict a cross section a factor 1.5–2 larger than the data, overestimating the measured cross section by more than 3 standard deviations. So does the prediction based on the HKN07 parametrization, which includes less gluon shadowing than EPS09.

The model AB-EPS08, significantly underestimates the measured cross section by about a factor of two (about 5 standard deviations), indicating that the gluon shadowing is too strong in the EPS08 parameterization. The leading twist calculation (RSZ-LTA) is also significantly below the data, by about 2–3 sigma.

For the incoherent cross section, shown in Fig. 6(b), there are three model predictions available, LM, STARLIGHT, and RSZ-LTA. The measured value deviates by about two standard deviations from the LM prediction, while STARLIGHT predicts an incoherent cross section 60 % too high, and RSZ-LTA a factor 4 too low. Taking the ratio between the incoherent and coherent cross section provides further constraints on the treatment of the nuclear modifications implemented in the different models. Another advantage is that the photon spectrum is factorized out, so that the comparison directly probes the ratio of the photonuclear cross sections. The ratio obtained from data is $0.41^{+0.10}_{-0.08} (\mathrm{sta+sys} )$. This can be compared with 0.21 from LM, 0.41 from STARLIGHT, and 0.17 from RSZ-LTA. Although the RSZ-LTA model is quite close for the coherent cross section at mid-rapidity, it seems to underpredict the incoherent cross section. The LM model also predicts a too low ratio. STARLIGHT, on the other hand, has about the right ratio of incoherent-to-coherent cross section, although it does not reproduce any of the cross sections individually. All three models use the Glauber model to calculate the incoherent cross section, but the implementation and the input cross section for *γ*+*p*→J/*ψ*+*p* varies. In STARLIGHT the scaling of the inelastic J/*ψ*+nucleus cross section, ranges from *A* ^2/3^ to *A*, depending on the J/*ψ*+nucleon cross section. In the first case, only the nucleons on the surface participate in the scattering, while in the second one all the nucleons contribute. The cross section for incoherent photoproduction is assumed in STARLIGHT to follow the same scaling, while in the other models, the reduction with respect to the *A* scaling is larger.

The measured values for the *γγ* cross sections are 20 % above but fully compatible within 1.0 and 1.5 sigma with the STARLIGHT prediction for the high and low invariant mass intervals, respectively, if the statistical and systematic errors are added in quadrature. This result provides important constraints on calculations that include terms of higher orders in *α*_em_. A reduction in the two-photon cross section of up to 30 % compared with leading-order calculations has been predicted [14, 15]. The result for the two-photon cross section to di-lepton pairs, measured by ALICE with a precision of 12 % and 16 % for the low and high invariant mass range, respectively, is thus fully consistent with STARLIGHT, and sets limits on the contribution from higher-order terms [16]. This result supports the ALICE J/*ψ* photoproduction measurement in the forward rapidity region [6], where the cross section was based on *σ*_*γγ*_.

## Summary

In summary, the first measurement of coherent and incoherent J/*ψ* photoproduction and two-photon production of di-lepton pairs in Pb–Pb collisions at mid-rapidity at the LHC has been presented and compared with model calculations. The J/*ψ* photoproduction cross sections provide a powerful tool to constrain the nuclear gluon shadowing in the region *x*≈10^−3^. The coherent J/*ψ* cross section is found to be in good agreement with the model which incorporates the nuclear gluon shadowing according to the EPS09 parameterization (AB-EPS09).

Models which include no nuclear gluon shadowing are inconsistent with the measured results, as those which use the Glauber model to incorporate nuclear effects. The AB-HKN07 and AB-EPS08 models contain too little or too much shadowing, respectively, to match the data. Our results are about 3 sigma higher than the RSZ-LTA model prediction, although a deviation of just 1.5 sigma is found from the model upper limit. Nevertheless the above predictions may have large uncertainties coming not only from the parametrization of the nuclear gluon distribution but also from the selection of the hard scale, the contributions from the higher-order terms and the treatment of the photon fluctuation to a quark–antiquark pair. The current measurement will contribute to resolve these uncertainties.

None of the three existing models predicts the incoherent photoproduction cross section correctly, but STARLIGHT predicts a correct incoherent-to-coherent ratio.

Finally, the measured two-photon cross section for di-electron production is consistent with the STARLIGHT model. This implies the models predicting a strong contribution of higher-order terms (not included in STARLIGHT) to the cross section are not favored.
